# Angiotensin II increases gene expression after selective intra-arterial adenovirus delivery in a rabbit model assessed using in vivo SSTR2-based reporter imaging

**DOI:** 10.1186/s13550-016-0183-x

**Published:** 2016-03-17

**Authors:** Sheela P. Singh, Murali K. Ravoori, Katherine A. Dixon, Lin Han, Sanjay Gupta, Rajesh Uthamanthil, Kenneth C. Wright, Vikas Kundra

**Affiliations:** Department of Cancer Systems Imaging, The University of Texas MD Anderson Cancer Center, 1515 Holcombe Blvd, Houston, TX 77030 USA; Department of Interventional Radiology, The University of Texas MD Anderson Cancer Center, 1515 Holcombe Blvd, Houston, TX 77030 USA; Department of Veterinary Medicine and Surgery, The University of Texas MD Anderson Cancer Center, 1515 Holcombe Blvd, Houston, TX 77030 USA; Department of Diagnostic Radiology, The University of Texas MD Anderson Cancer Center, 1515 Holcombe Blvd, Houston, TX 77030 USA; UT MD Anderson Cancer Center, 1400 Pressler St., Unit 1473, Houston, TX 77030 USA

**Keywords:** Gene therapy, Gene delivery, Angiotensin II, Somatostatin receptor type 2, Imaging

## Abstract

**Background:**

Gene therapy has been hampered by low expression upon in vivo delivery. Using a somatostatin receptor type 2 (SSTR2)-based reporter, we assessed whether angiotensin II (AII) can improve gene expression by adenovirus upon intra-arterial (IA) delivery in a large animal model.

**Methods:**

A SSTR2-based reporter that can be imaged by a clinically approved radiopharmaceutical was used to assess gene expression. Eight rabbits bearing VX2 tumors in each thigh were randomly injected IA with adenovirus containing a human SSTR2 (Ad-CMV-HA-SSTR2) gene chimera ± AII or control adenovirus containing green fluorescent protein (Ad-CMV-GFP). Three days later, ^111^In-octreotide was given IV after computed tomography (CT) imaging using a clinical CT scanner and intravenous contrast. Tumor uptake of ^111^In-octreotide was evaluated the next day using a clinical gamma camera. Gene expression was normalized to tumor weight and morphology from CT to obtain in vivo biodistribution.

**Results:**

SSTR2-based expression was readily visualized. VX2 tumors infected with Ad-CMV-HA-SSTR2 upon intra-arterial delivery with AII had greater in vivo biodistribution, thus greater gene expression, than those without AII (*p* < 0.01, *n* = 6). VX2 tumors infected with Ad-CMV-HA-SSTR2 upon IA delivery had greater biodistribution, thus greater gene expression, than those with the negative control Ad-CMV-GFP (*p* < 0.02). Similarly, VX2 tumors infected with Ad-CMV-HA-SSTR2 upon IA delivery with AII had greater biodistribution, thus greater gene expression, than those with the negative control Ad-CMV-GFP (*p* < 0.01).

**Conclusions:**

Angiotensin II improves in vivo gene expression by adenovirus upon intra-arterial delivery and thus may improve gene therapy efficacy. In vivo SSTR2-based reporter imaging can be used to compare methodologies for improving gene expression.

## Background

Two of the major limitations to gene therapy have been delivery and an inability to monitor exogenous gene expression in vivo. For the former, the vector may be delivered by direct injection into the parenchyma of the tumor or organ of interest, via inhalation, intravenously, or intra-arterially. Systemically, venous delivery requires first pass through the lungs and/or the liver, where the vector may become entrapped or infect; thus, much of the vector, such as commonly clinically used adenovirus, may not be delivered to the tumor or organ of interest and expression may occur in the wrong location for efficacy. Arterial delivery may be used to direct the vector to the organ or tumor of interest, particularly when delivery is directed via a catheter to the primary artery feeding the tumor. Even with this approach, expression may not be greater than intra-tumoral delivery [1]. Whether pharmacologic manipulation can increase expression upon intra-arterial adenovirus delivery needs exploration.

Numerous vasoactive agents have been evaluated using pharmacoangiography to modify tumor blood flow in an effort to improve diagnosis and treatment [2, 3]. Of the vasoconstrictors tested, angiotensin II (AII) was found superior diagnostically, especially for poorly vascularized tumors supplied by small arteries [2]. Subsequently, selective arterial infusion of AII was investigated as a way to maximize tumor delivery of chemotherapeutic agents and minimize distribution to non-tumor regions [4–8]. Since blood vessels in tumors, including VX2 rabbit tumors, commonly lack smooth muscle [9, 10], theoretically, arterial infusion of vasoconstricting agents should constrict normal vessels but leave tumor vessels unaffected, shunting blood from normal tissues to tumor. We recently demonstrated that at an optimal dose of 2.5 μg/ml, AII increases perfusion of VX2 tumor versus normal tissue by increasing tumor blood flow and blood volume but decreases mean transit time [11]. Whether angiotensin II can increase expression upon intra-arterial adenovirus delivery needs further evaluation.

To address such questions and to monitor gene therapy, there is a need to noninvasively locate, quantify, and longitudinally assess expression after delivery. Clinically, exogenous gene expression is most commonly followed by biopsy; however, the entire lesion or organ is not evaluated, there is potential for sampling error, and repeated analysis is difficult because of the potential for adverse complications and patient compliance. Noninvasive imaging presents a means to overcome these problems by monitoring and quantifying gene expression through the use of a reporter-based system. Reporter genes commonly encode enzymes, transporters, or receptors [12–18] which entrap a radiopharmaceutical that can be used for imaging.

Human somatostatin receptor type 2 (SSTR2)-based reporter systems can be used to visualize exogenous gene expression. We have demonstrated that a combination of anatomic and functional imaging can be used to quantify SSTR2-based gene expression [18–20] in small animal models using the FDA-approved radiopharmaceutical ^111^In-octreotide. Such models were primarily studied using dedicated small animal equipment. In such models, the small vessel size restricts catheter-based access and vascular manipulation. The large animal VX2 rabbit tumor model allows catheterization [1] using human catheters, vascular manipulation, and imaging using clinical machines and thus can be used to study the effect of pharmacologic vascular manipulation like AII on gene expression. Gene therapy has been hampered by low levels of gene expression upon in vivo delivery. Using a SSTR2-based reporter, we assessed whether angiotensin II can improve gene expression by adenovirus upon intra-arterial delivery in a rabbit VX2 tumor model.

## Methods

### Adenoviruses

Ad-CMV-HA-SSTR2 was constructed and expression was confirmed by in vitro experiments as previously described [18]. The control adenovirus contained a CMV promoter with green fluorescent protein (Ad-CMV-GFP). Both viruses were purified and amplified by the Vector Core lab at UT MD Anderson Cancer Center. VX2 tumors are carried as tumor implants and not as cell lines. Immunofluorescence imaging was performed using HT1080 fibrosarcoma cells (ATCC, Rockville, MD) as described [21].

### Animal and tumor model

Nine New Zealand white rabbits (Myrtle’s Rabbitry, Thompsons Station, TN) weighing 3 to 4.5 kg and rabbit VX2 carcinoma cells were used in this study. The VX2 cells were grown by implanting into the hind limb of several donor rabbits. When the resulting tumors reached 2 cm in diameter, the tumors were freshly harvested under sterile conditions, minced, and homogenized in DMEM/F12 containing 10 % FBS (Media Tech Inc., Manassas, VA) and gentamicin. The tumor fragments were aliquoted into 12 × 75 mm plastic tubes and stored at −80 °C until further use [11]. When needed, they were thawed, suspended in DMEM/F12 containing 10 % FBS and gentamicin, and then drawn into a 1-cc syringe with an 18-gauge needle. A total of 0.6 ml of homogenized VX2 tumor cells were implanted in both thighs of each experimental rabbit [1]. All animals received humane care in compliance with the Institutional Animal Care and Use Committee of The University of Texas MD Anderson Cancer Center.

### Imaging and biodistribution

Nine adult New Zealand white rabbits were used to assess reporter gene expression in VX2 carcinoma after intra-arterial (IA) in vivo gene transfer. The VX2 carcinoma was implanted intramuscularly into both thighs of each animal (18 tumors total). Unfortunately, one rabbit died for unknown reasons after tumor implantation. A total of 16 tumors from eight rabbits were utilized for this study. After 10–12 days, multi-slice helical computed tomography (CT) imaging without and with intravenous contrast enhancement was performed in a supine position to confirm tumor formation and to calculate tumor weight and volume [1].

Each tumor was assigned an adenovirus delivery plus or minus AII. Six tumors underwent IA delivery (see below) of AII and Ad-CMV-HA-SSTR2, six with Ad-CMV-HA-SSTR2, and four tumors with Ad-CMV-GFP as control (100 μl each, MOI 10). Tumors in the same animal received any two of the above treatments, i.e., one treatment in the right leg tumor and a different treatment in the left leg tumor. Three days later, CT imaging was repeated and the animals were injected via a marginal ear vein with 22 MBq (600 μCi) of ^111^In-octreotide (Octreoscan, Mallinckrodt, St. Louis, MO). The following day, planar gamma camera imaging was performed. The amount of ^111^In-octreotide in each tumor was determined from the planar images and normalized to the tumor weight (%ID/g) calculated from the CT images [1]. After imaging, the rabbits were sacrificed with an intravenous overdose of Beuthanasia-D. Portions of tumor or organs were dissected and weighed; the amount of radioactivity was measured using a γ-counter to determine ^111^In-octreotide biodistribution in %ID/g.

### Intra-arterial route of injection

Vascular access was obtained as described [1]. Briefly, each rabbit was sedated using isoflurane (5 %)/oxygen (1.5 l/min) via mask administration. An endotracheal tube was inserted, and anesthesia was maintained with isoflurane (3–4 %)/oxygen (1.5 l/min). The antibiotic Enrofloxacin (Baytril; Bayer Corp; Agriculture Division, Animal Health, Shawnee Mission, KS) was given intramuscularly (5 mg/kg), and the neck was shaved and prepared for aseptic surgery using alcohol and Betadine scrub. A small midline incision was made to isolate and cranially ligate the right common carotid artery. Proximal to the ligation, a small arteriotomy was made to insert a 4.0-F sheath (Cook Incorporated, Bloomington, IN) into the vessel, where sodium heparin (100 IU/kg) was administered. A 2.8-F microcatheter (EmboCath; BioSphere Medical, Rockland, MA) was inserted through the sheath and directed down the aorta to its bifurcation using fluoroscopic monitoring. The catheter was then manipulated into each external iliac artery. In order to assess vascular anatomy and tumor location, digital subtraction arteriography was performed by injecting radiographic contrast medium, meglumine-diatrizoate (Conray 60 %; Mallinckrodt Inc., St Louis, MO), and by hand using fluoroscopic guidance. The microcatheter was then positioned into the deep femoral artery, which is the main, if not exclusive, artery supplying the tumor. The catheter was flushed with saline first, and then 100 μl (MOI of 10) of adenovirus with or without 2.5 μg/ml angiotensin II (GenScript USA, Piscataway, NJ) in 10 ml saline was slowly injected; the catheter was flushed once again with saline. Angiotensin II has an approximately 15-s half-life [22]. The catheter was then manipulated into the deep femoral artery in the opposite thigh, and the process was repeated. Then, the catheter and introducer sheath were removed, the carotid artery was ligated proximal to the arteriotomy, and the incision was closed in two layers.

### Computed tomography imaging

All multi-slice helical computed tomography (CT) (Lightspeed Plus; GE Medical Systems, Milwaukee, WI) imaging was obtained using the following parameters: 120-kVp tube voltage, 80-mA tube current, 25-cm field of view, and 1.25-mm slice thickness. For contrast-enhanced CT imaging, 8.0 ml of Visipaque 320 contrast medium (iodixanol; GE Healthcare, Inc., Princeton, NJ) was injected through a marginal ear vein at a rate of 1.5 ml/s. After a 9-s delay, CT images of the thighs were acquired.

In order to determine tumor volume and weight, a region of interest (ROI) was drawn around the entire tumor as visualized on the CT images with or without contrast enhancement. Tumor volume was calculated as previously described [1] using volume viewer application software for CT (Voxtool 3.0.64z, GE Medical Systems). Assuming a tumor density of 1.0 g/ml, tumor volume (cm^3^) was converted to tumor weight. A similar process was used to trace and calculate the weight of necrotic tissue within the tumor images identified as tumor areas that did not enhance after contrast administration. The weight of the necrotic tissue was then subtracted from the tumor weight to calculate the weight of the tumor without necrosis [1, 20].

### Planar gamma camera imaging

The rabbits were positioned 2 cm directly below a medium-energy parallel-hole collimator [1]. Planar imaging was performed for 30 min with a clinical flexible single-head gamma camera (Digirad Corporation, Poway, CA). The resulting images were transferred and processed in DICOM format to an eSoft workstation. For each animal, a ROI of the same size was drawn on both the tumor and background regions to determine the average counts per pixel; radiopharmaceutical uptake by tumors in microCi were calculated from correlation equations derived from microCi versus counts per pixel graphs created using phantoms containing different amounts of ^111^In-octreotide in a total volume of 500 μl [20].

### Western blot

Proteins extracted from tumors infected IA with and without AII-Ad-CMV-HA-SSTR2 or Ad-CMV-GFP control were used to perform western blotting [1] using a mouse-anti HA antibody (Covance, Princeton, NJ) at 1:500 dilution and a secondary goat anti-mouse antibody (Santa Cruz, Santa Cruz, CA) at 1:3000 dilution.

### Statistical analysis

A power calculation determined that with alpha = .05, a sample size of 4 would provide >80 % power to compare groups. The data are presented as mean ± standard deviation. Two-tail Student’s *t* test was used for making comparisons between the groups and were analyzed using Microsoft Excel 2007 (Microsoft Corp., Redmond, WA). The primary comparison was Ad-CMV-HA-SSTR2 plus versus minus AII. *p* < 0.05 was considered statistically significant.

## Results

### Ad-CMV-HA-SSTR2 results in HA-SSTR2 expression in vitro

**Fig. 1:**
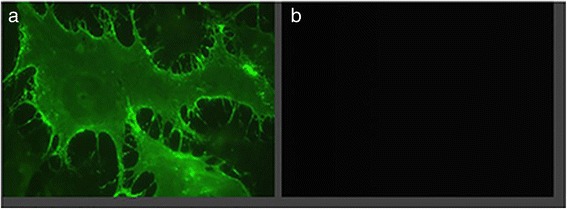
Immunofluorescence. Indirect immunofluorescence demonstrates fluorescence, thus hSSTR2 expression, in cells infected with **a** Ad-CMV-hSSTR2 but not in the **b** uninfected negative control

The VX2 tumors are carried as tumor implants and not as cell lines. To confirm virus expression of HA-SSTR2, HT1080 cell lines were used. Immunofluorescence imaging demonstrated expression of HA-SSTR2 on the cell membrane as expected (Fig. 1). In comparison, no expression was seen in the control uninfected cells. This is consistent with previous findings demonstrating HA-SSTR2 expression with this virus [18].

### Angiotensin II results in increased blood flow to the tumor

**Fig. 2:**
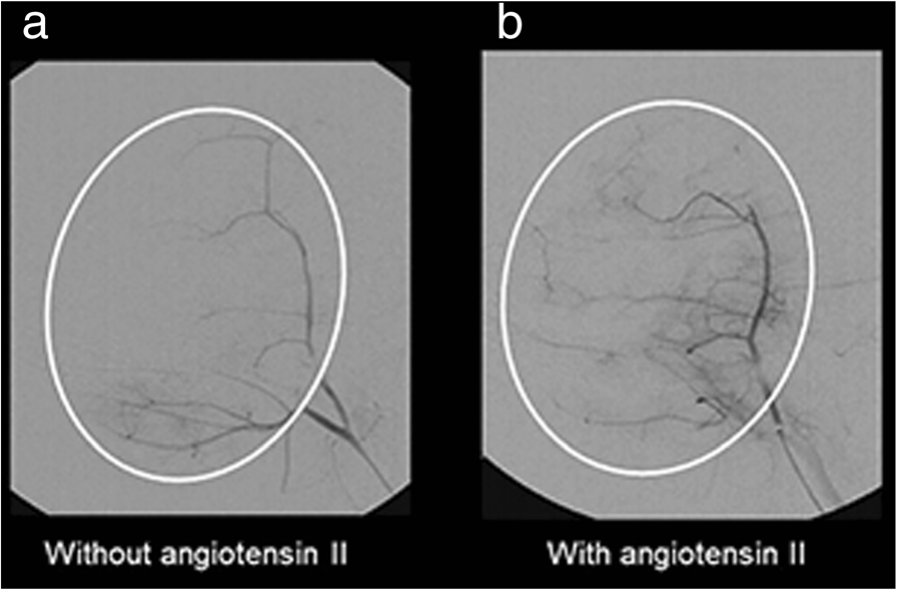
Angiography. Representative fluoroscopic images. The vasoconstrictor angiotensin II (2.5 μg/ml) was administered through the femoral artery to the tumor. Baseline blood flow was seen before AII injection (**a**). A “tumor blush” consistent with increased tumor blood flow was seen after AII administration (**b**)

Infusion of the vasoconstrictor AII at a dose of 2.5 μg/ml into the vessel resulted in visualization of more vessels at fluoroscopy, when compared to the no AII (Fig. 2). The “tumor blush” is consistent with increased blood flow to the tumor upon AII infusion and is consistent with our previous findings suggesting increased tumor blood flow and tumor blood volume upon angiotensin II exposure at 2.5 μg/ml [11].

### Morphological assessment of reporter gene expression after in vivo gene transfer

**Fig. 3:**
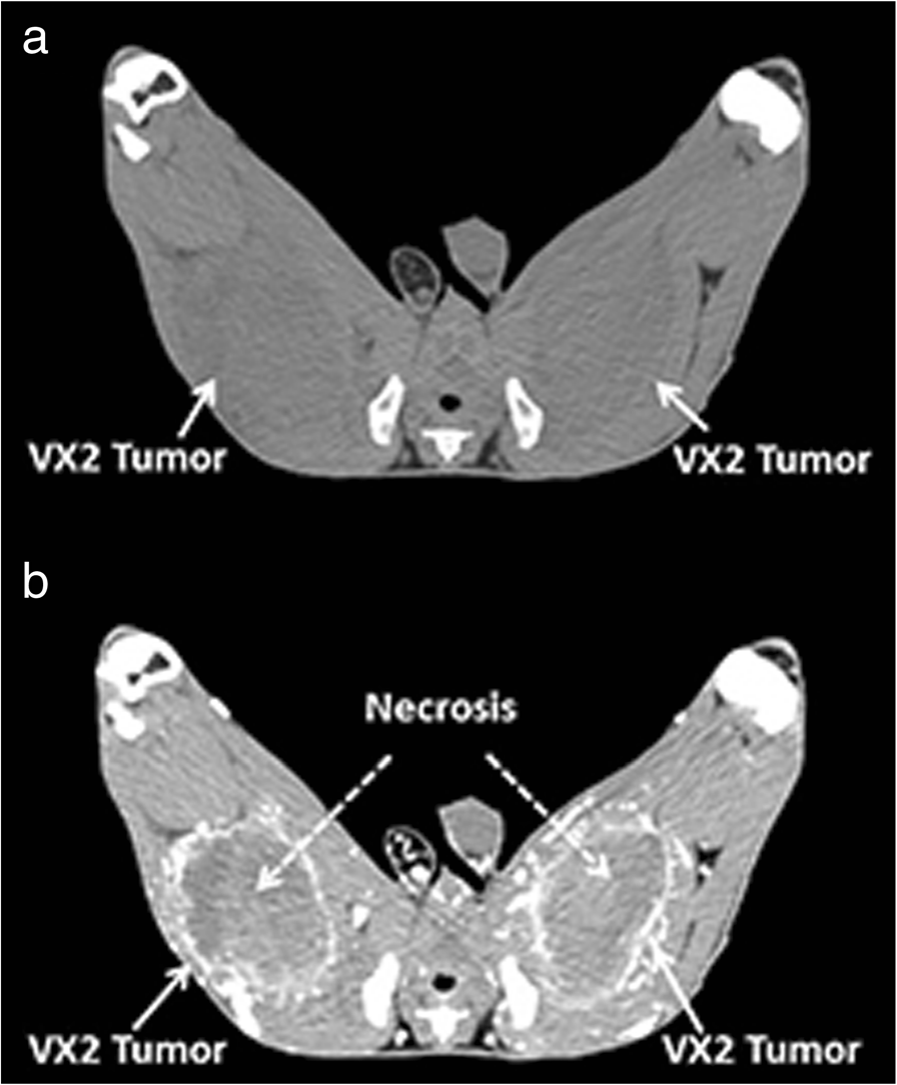
Representative transverse in vivo CT images of a rabbit hind leg with VX2 tumors. **a** Unenhanced (without contrast) CT image acquired 2 weeks after adenovirus injection. Tumors appear as areas of low attenuation (*arrows*) and are relatively difficult to visualize. **b** Contrast-enhanced CT image showing well-defined peripheral rim enhancement of each tumor (*solid white arrows*). Areas of unenhanced necrosis (central low attenuation material) are seen inside the tumor, as indicated by the *dotted white arrows*

Three days after adenovirus infection, representative axial CT images of VX2 tumors were obtained with and without intravenous contrast enhancement (Fig. 3). In vivo unenhanced CT imaging demonstrated areas of low attenuation, but areas of necrosis could not be distinguished (Fig. 3a). On the other hand, contrast-enhanced imaging (Fig. 3b) illustrated peripheral rim enhancement of live tumor (13.49 ± 4.85 g for Ad-CMV-HA-SSTR2 + A II, 15.49 ± 8.37 g for Ad-CMV-HA-SSTR2, and 14.99 ± 7.64 g for Ad-CMV-GFP; mean ± SD) and lack of enhancement of the necrotic center (22.61 ± 6.94 g for Ad-CMV-HA-SSTR2 + A II, 27.38 ± 21.21 g for Ad-CMV-HA-SSTR2, and 14.50 ± 1.29 g for Ad-CMV-GFP; mean ± SD). The total tumor weights derived from in vivo enhanced CT imaging (36.20 ± 7.21 g for Ad-CMV-HA-SSTR2 + A II, 42.87 ± 24.84 g for Ad-CMV-HA-SSTR2, and 29.48 ± 8.54 g for Ad-CMV-GFP; mean ± SD) correlated well with the weights of excised tumors (*r* = 0.97, *n* = 16)

### In vivo assessment of reporter gene (Ad-CMV-HA-SSTR2) expression in VX2 tumors after gene transfer with angiotensin II

**Fig. 4:**
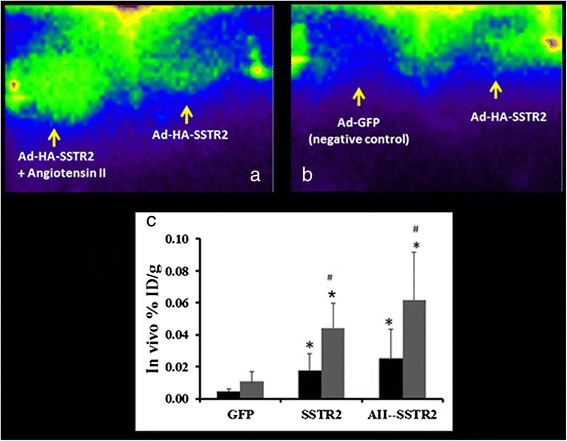
γ-Camera imaging data of VX2 tumors in rabbits. Representative gamma camera planar images of VX2 tumors (**a**) infected in vivo by IA administration of AII + Ad-CMV-HA-SSTR2 and Ad-CMV-HA-SSTR2 and (**b**) infected in vivo by IA infusion of Ad-CMV-GFP control and Ad-CMV-HA-SSTR2 show increased ^111^In-octreotide uptake in tumors infected with AII + Ad-CMV-HA-SSTR2 compared to infection with Ad-CMV-HA-SSTR2 and the control Ad-CMV-GFP. **c** ^111^In-octreotide biodistribution in tumors normalized to tumor weight (%ID/g) calculated with (*black bars*) and without (*gray bars*) necrosis using in vivo imaging from gamma camera and CT imaging. Uptake was higher in tumors infected with AII + Ad-CMV-HA-SSTR2 (*n* = 6) compared to control Ad-CMV-GFP (*p < 0.01, *n* = 4) and Ad-CMV-HA-SSTR2 (*p < 0.01, *n* = 6). The %ID/g without necrosis was significantly higher than the %ID/g with necrosis in tumors infected with AII + Ad-CMV-HA-SSTR2 (#*p* < 0.004, *n* = 6) and Ad-CMV-HA-SSTR2 (#*p* < 0.006, *n* = 6)

Three days after adenovirus infection, the animals were injected via a marginal ear vein with ^111^In-octreotide, which binds SSTR2. The following day, planar gamma camera imaging (Fig. 4) demonstrated increased uptake in tumors infected with Ad-CMV-HA-SSTR2 compared to negative control Ad-CMV-GFP, and uptake appeared greater if AII was also given with Ad-HA-SSTR2 compared to Ad-HA-SSTR2 alone (Fig. 4). In vivo biodistribution using a combination of CT and gamma camera imaging demonstrated significantly greater uptake of ^111^In-octreotide in VX2 tumors infected with AII plus Ad-CMV-HA-SSTR2 (0.025 ± 0.018; %ID/g mean ± SD) compared to Ad-CMV-HA-SSTR2 (0.018 ± 0.011, %ID/g mean ± SD) (*p* < 0.01; *n* = 6) alone or negative control Ad-CMV-GFP (0.005 ± 006; %ID/g mean ± SD) (*p* < 0.01; *n* = 6 AII plus Ad-CMV-HA-SSTR2, *n* = 4 Ad-CMV-GFP, Fig. 4). Uptake was significantly higher in tumors infected with Ad-CMV-HA-SSTR2 alone versus Ad-CMV-GFP (*p* < 0.004, *n* = 6 and *n* = 4), respectively. Necrotic regions of dead cells should not express genes, and consistent with this, we have shown previously that background uptake is found in areas of necrosis [1]. The %ID/g without necrosis (areas of live/perfused tissue) was significantly higher than the %ID/g with necrosis in tumors infected with AII plus Ad-CMV-HA-SSTR2 (0.062 ± 0.03; %ID/g mean ± SD) (*p* = 0.004; *n* = 6) and Ad-CMV-HA-SSTR2 (0.044 ± 0.016, %ID/g mean ± SD) (*p* = 0.006; *n* = 6) (Fig. 4c). No significant change was observed in the animals injected with negative control Ad-CMV-GFP (0.011 ± 0.006; %ID/g mean ± SD).

**Fig. 5:**
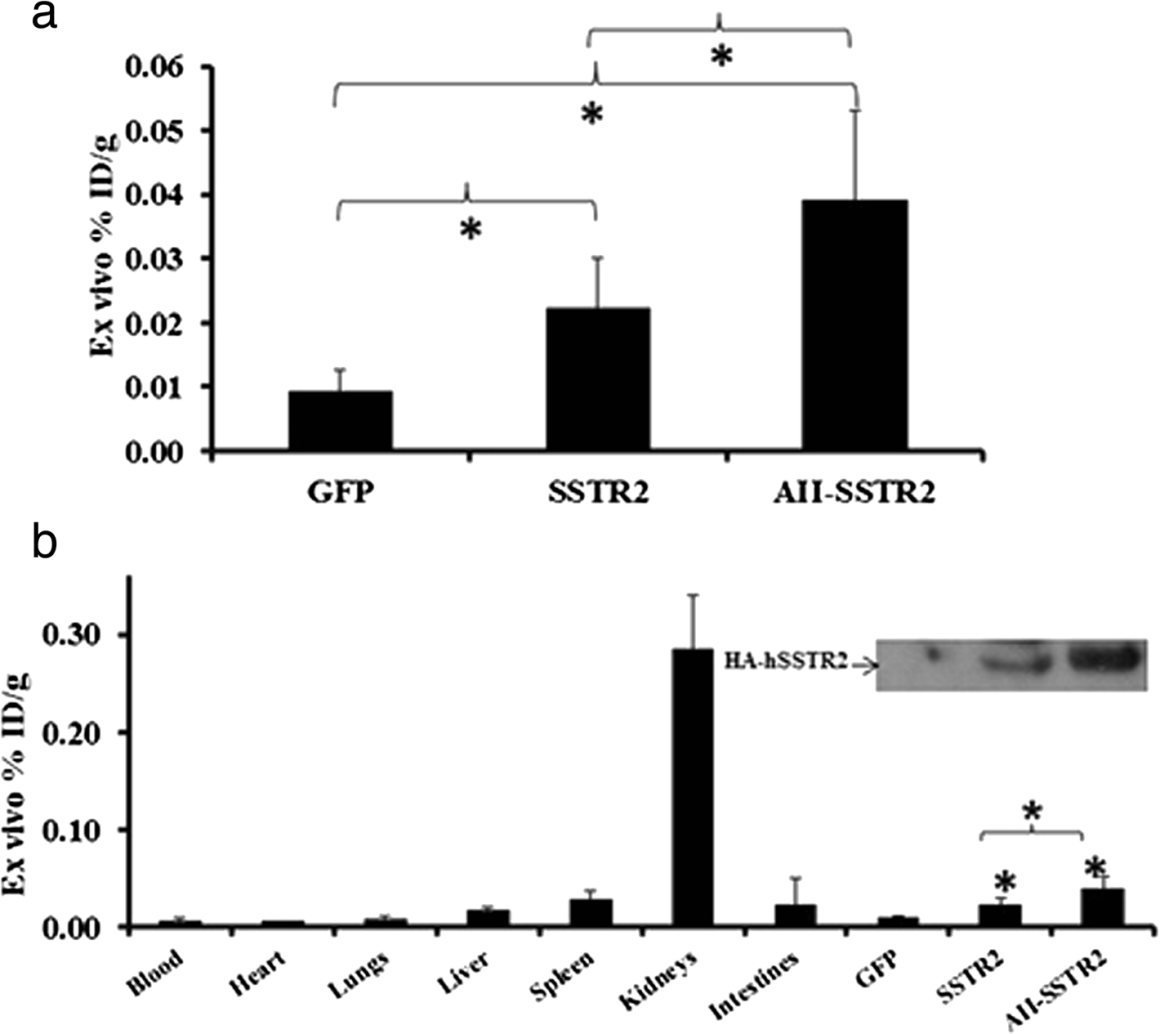
Ex vivo analysis of ^111^In-octreotide biodistribution in VX2 tumors after adenoviral infection. **a** Graph showing significantly higher uptake in tumors infected with AII + Ad-CMV-HA-SSTR2 compared to tumors infected with control Ad-CMV-GFP (*p < 0.004, *n* = 4) and Ad-CMV-HA-SSTR2 (*p < 0.002, *n* = 6). The ex vivo %ID/g of Ad-CMV-HA-SSTR2 was also significantly higher compared to that of control Ad-CMV-GFP (*p* = 0.015). **b** Graph showing ex vivo organs and tumor biodistribution of ^111^In-octreotide in rabbits bearing VX2 tumors infected in vivo with AII + Ad-CMV-HA-SSTR2, Ad-CMV-HA-SSTR2, or control virus. Increased uptake was seen in tumors infected with AII + Ad-CMV-HA-SSTR2 or Ad-CMV-HA-SSTR2 as compared to tumors infected with control Ad-CMV-GFP (*p < 0.015, *n* = 4), which was also found by western blot analysis of HA-SSTR2 in tumors (*inset*)

Ex vivo evaluation of the radiotracer biodistribution demonstrated significantly higher levels of ^111^In-octreotide in tumors infected with AII plus Ad-CMV-HA-SSTR2 (0.039 ± 0.014; %ID/g mean ± SD) compared to tumors infected with Ad-CMV-HA-SSTR2 (0.022 ± 0.008, %ID/g mean ± SD) (*p* = 0.002; *n* = 6) or negative control Ad-CMV-GFP (0.009 ± 0.0032; %ID/g mean ± SD) (*p* = 0.004; *n* = 6 AII plus Ad-CMV-HA-SSTR2, *n* = 4 Ad-GFP); ex vivo %ID/g of Ad-CMV-HA-SSTR2 was also significantly greater compared to control Ad-CMV-GFP (*p* = 0.015; *n* = 6 Ad-CMV-HA-SSTR2, *n* = 4 Ad-GFP) (Fig. 5a). This corresponded to in vivo biodistribution findings. Thus, administration of AII significantly increased tumor transgene expression. As expected, the kidneys exhibited the highest uptake of ^111^In-octreotide compared to other organs since the normal route of excretion for the radioligand is primarily through the kidneys with secondary routes via the liver and the intestines (Fig. 5b). Western blot for the HA tag showed HA-SSTR2 expression in tumors infected with Ad-CMV-HA-SSTR2. Tumors infected with AII plus Ad-CMV-SSTR2 showed the highest HA-SSTR2 expression (top panel Fig. 5b).

## Discussion

Clinical trials of gene therapy most commonly target cancer and most commonly utilize adenovirus as the vector [23]. Two of the major limitations for gene therapy are delivery and an inability to monitor exogenous gene expression in vivo. Using a SSTR2-based reporter system, we demonstrate that angiotensin II can be used to increase gene expression upon intra-arterial adenovirus delivery. For these experiments, a large animal model was utilized as a bridge to translation since it enabled the use of catheterization techniques and imaging equipment employed in the clinic. For imaging the reporter, an FDA-approved radiopharmaceutical was used. To the best of our knowledge, this is the first study to demonstrate the efficacy of angiotensin II in improving gene expression upon intra-arterial delivery of adenovirus in a large animal model.

The use of a large animal model in this study allowed for selective vascular access, enabling vascular manipulation for augmenting intra-arterial gene delivery. This is possible because the catheter can be manipulated under fluoroscopic image guidance into the artery feeding the tumor for injecting the adenovirus. This type of vascular access is difficult to perform in mouse models due to the smaller vessel size. IA delivery is already commonly performed in patients, for example, for those suffering from arteriovenous malformation (AVM), liver metastases, muscle disease, or brain tumors.

A vector may be delivered via several routes such as by direct injection into the tumor, via inhalation, intravenously, or intra-arterially. Gnant et al. [24] showed tumor-specific gene delivery is possible after systemic injection of a thymidine kinase-negative vaccinia virus vector in a rabbit liver metastasis model. However, systemic delivery such as via the venous system can result in vector loss due to first pass through organs such as the lungs and the liver, where the vector may become entrapped or infect. Intra-arterial delivery may be used to direct the vector to the tumor. For example, Barnett et al. [25] performed adenoviral intra-arterial gene delivery of anti-angiogenic endostatin in a syngeneic rat gliosarcoma model and demonstrated transgene expression and therapeutic efficacy. Similarly, Geschwind et al. [26] directed the ATP production inhibitor, 3-bromopyruvate, into liver-transplanted rabbit VX2 tumors via the IA delivery route and inhibited tumor growth. Soel et al. [27] demonstrated in vivo transgene expression after IA delivery of a DNA/liposome/transferrin complex into VX2 tumors in rabbit livers using β-galactosidase staining. On the other hand, Kim et al. [28] evaluated the feasibility of an iodized oil emulsion system that can be used for chemoembolization of hepatocellular carcinoma as a modifier of non-viral gene transfer for IA gene delivery in hepatic tumors. Although these studies support the therapeutic success of IA gene delivery, in vivo quantification of gene expression was not evaluated. Gene therapy via the IA route has been proposed as a better alternative to other routes, such as intra-tumoral injection, because IA delivery should allow for repeated dosing and widespread distribution within the tumor [11]. However, there have been reports to the contrary, finding IA and IT routes leads to similar levels of expression [1].

For the first time, we were able to both in vivo quantify and improve expression upon adenovirus delivery using angiotensin II. Fluoroscopic imaging demonstrated increased tumor blush consistent with increased blood flow to the tumor upon delivery with AII. This suggests improved adenovirus delivery. We previously found in a liver model that a 2.5 μg/ml dose of AII increased blood flow, blood volume, and permeability surface product suggesting that a strategy employing AII may improve delivery; however, it also decreased the mean transit time in VX2 tumors compared to normal tissue [11]. The current study demonstrates that such a strategy employing AII can result in improved expression after adenovirus delivery. Our study is supported by Bilbao et al. who using adenovirus with a lacZ insert noted AII at one dose could increase lacZ expression detected by histology in larger rat hepatomas but not smaller hepatomas [29].

Our previous data that at the appropriate dose, AII leads to a tumor blush with poorer delineation of small vessels, consistent with their vasoconstriction, and dynamic contrast-enhanced imaging data demonstrating that blood flow was diverted to the tumor [11] is consistent with the proposed mechanism of action of AII causing vasoconstriction of normal vessel arterioles but not the abnormal vessels in tumors that lack functional arterioles and have decreased number of AII receptors [30]. This would preferentially lead to increased blood flow and blood volume for adenovirus delivery; these effects and increased permeability appear to be more significant than the decreased mean transit time noted on the prior study DCE-CT study [11]. Given that the mechanism of action is via vascular manipulation, we predict that the AII strategy is likely applicable to other gene therapy vectors for improving gene delivery and expression to various sites in the body. For evaluating strategies for improving gene expression, in vivo reporter imaging technologies with potential for human use, such as those that are SSTR2-based, are useful.

Exogenous HA-SSTR2 gene expression can be quantified in a mouse model using functional and anatomic imaging [18–20]. Large animal models not only enable vascular manipulation like in humans but also can grow larger tumors with necrosis—as is found in patients. Necrosis occurs in solid tumors in the setting of insufficient blood supply during the natural history of the disease or as a response to therapy. In a large animal model, we found that necrosis could be identified on contrast-enhanced CT and does not pool the radiopharmaceutical, rather only background uptake was noted [1]. Areas of necrosis should not express the transferred gene. Thus, in the present study, necrotic areas were excluded from calculations of %ID/g, resulting in corrected, increased expression in the remaining live tumor. Ex vivo analysis confirmed increased expression upon intra-arterial AII delivery with adenovirus delivery. Anatomic and functional imaging also enables longitudinal quantification of expression upon SSTR2-based gene delivery [1, 18]. Current findings further support the use of SSTR2-based reporters for assessing methods to improve gene delivery and expression with the ultimate goal of improving gene therapy efficacy. The use of novel reporters, such as signaling deficient-SSTR2, has prospective utility since they are limited in their effects on normal cell signaling and cellular responses, such as replication [31]. Moreover, expression of potential therapeutic genes may be evaluated by linking them to SSTR2-based reporters. Thus, methods to noninvasively monitor gene transfer using SSTR2-based reporters may have wide applicability towards improving and assessing gene therapy; further example applications may include vector development, assessing promoter activity, and designing dosing regimens.

## Conclusions

In summary, our study demonstrates improved gene expression when using angiotensin II in combination with adenovirus upon intra-arterial delivery in a large animal model. Gene expression was measured using a SSTR2-based reporter system that was visualized using clinical machines and an FDA-approved radiopharmaceutical. Thus, this paper addresses two fundamental needs of gene therapy, improved delivery and monitoring of gene expression. The findings suggest that intra-arterial delivery of angiotensin II with adenovirus augments expression and thus may improve gene therapy efficacy and that SSTR2-based reporter imaging is useful in comparing methods for enhancing gene expression in vivo.
